# Among the Out-Patients

**Published:** 1887-02-12

**Authors:** 


### Among the Out-Patients.

By One of the Crowd.

The Men Uninteresting.

Taken as a whole, the men are a much less interesting study than the women; although, of course, many of the remarks I ventured to make in a former paper on the general appearance of the out-patients I have come into contact with, apply equally to both sexes. In every class of life the lords of creation are seen at a disadvantage when they are doing nothing, as compared with the so-called inferior half of humanity when they are similarly occupied. What more melancholy spectacle than a wall-flower in a ball-room, or the men who are dragged day after day to the crushes, facetiously called entertainments, of which the London season is mainly composed? An unemployed woman—one, that is, who is condemned to an enforced period of idle waiting—is in very different case; and, even if there is no one to talk to, she can interest herself by making mental comments upon the appearance of her neighbours. So in the out-patients' ward of the hospitals, whilst the women sit chattering, reading, knitting or sewing, the men on the other side of the room are so many rows of helpless taciturnity. Here and there a conversation may be proceeding, generally amongst the less dejected portion of the company, but it is carried on in a low tone, as if the speakers were afraid that the ghosts of departed out-patients might be flitting about the waiting-room. Having often heard that the chief complaint of out-patients has reference to this enforced period of waiting, I naturally endeavoured to find out how much truth there is in the statements as to the additional hardship thus inflicted upon the sick poor. Of course, some delay there must be, but at Guy's it has undoubtedly been reduced to a minimum. They have a system by which new patients attend an hour before those who have been once or more previously seen by the physician, or surgeon, as the case may be. These new-comers, too, undergo a sort of brief *viva voce* examination, as they wait in the outer hall, at the hands of a couple of gentlemen of the hospital staff, who are provided each day with a certain number of tickets. It goes without saying that the applicants are more numerous than the coveted pieces of paper, so the more pressing or more deserving cases are admitted, and the remainder are told to come on the next general day, when their claims will have preference. In this way, both the new and the old patients are benefited by the curtailment of the period of delay, and consequently the complaints of loss of time are very few at Guy's, as compared with some of the other hospitals.

The Weary Waiting.

There can be no doubt, however, that even one hour's detention is a very serious matter to some of the men who attend for treatment and medicine at our great healing charities; and perhaps it is this circumstance which is responsible for two things which have struck me. The first is the preponderance of women over men; the second is the apparently more serious nature of the ailments from which the majority of the men suffer. I am ignorant as to whether this be the experience of the Medical Officers or not. I speak as a layman solely from my own observation, and observations, as we all know, are occasionally misleading, sometimes, indeed, being altogether upset by statistics. But I am fortified in my opinion by a conversation I held with one very intelligent-looking man who was waiting to see the physician on the occasion of one of my visits. "Yes," he said, in answer to my inquiries, "it is more than an inconvenience for me to wait here, although I quite admit that they do their best to let the people get away as early as possible. In my case, it means the loss of money which I can ill afford. To-day, for instance, I lose just half my day's pay, and if it was not for the consideration of our master I might be turned off altogether." He proceeded to inform me that he was employed at a neighbouring wharf, and lived in the model buildings just below St. George's Church, on the other side of the road. "A good many men," he added, "who can at all afford the extra money, go to the provident dispensaries, not because they like the doctors so well, because they *don't*, but because they can go there of an evening after the work is done."—"Then why don't you do the same thing?" I asked. "I would," he answered, "and have done before, but my complaint"—he did not tell me what it was, and I didn't care to ask him—"is one which, I am told, requires the advice of a skilful and experienced doctor, and my wife, bless her! persuaded me to come here. 'Tom,' she said, 'the half a day lost every fortnight won't be so very much, and I'll try if I can't save it out of something or other,' so here I am." I expressed a hope that his attendance at the hospital would soon cease to be necessary, to which he gave a pious assent, and I left him, feeling in my own mind that here was just one of those cases which was peculiarly one for the aid of the out-patients' department of a hospital.

A Philosopher.

Quite a different person was my next interlocutor. He was standing in the Great Maze Pond—the thoroughfare n which the entrance for out-patients a Guy's is situated. I thought he was an Irishman, by his appearance, but it appeared he was only of Irish birth, London light having been the first to meet his eyes. He belonged to the unskilled labouring class, but his physique was poor, and his obvious stuggle with want had evidently been only a repetition of the experience of his parents and grand-parent. Yet he, too, was married and had some children, as he informed me; though what sort of men and women they were likely to become was only too evident. Giant London is a very tyrant, and uses up its poorest sons and daughters with a refinement of cruelty. Take this man, for instance. He did not lack stature, and had he been brought up in the fresh air of the country, might have been a hearty labourer worthy of his daily hire. But privation and close quarters, and unsanitary surroundings generally, had done their work, and the sons, unless somebody other than the parents gets hold of them, will assuredly be less able to fight the battle of life than their sire. Still, he was perfectly good-humoured, and was lounging against the great iron railings, whilst everybody else was crowding through the open door—for it was just two o'clock. He introduced himself, observing that I was eyeing him curiously, with a request for a pipe of tobacco, which, being complied with, let loose the tide of his garrulity. "Why don't you go in," I asked, "and not stand here smoking, which, by the way, won't do that cough of yours any good?" "Well," he said, "it's the very fur-r-st pipe"—his Hibernian ancestry came out now and again—"I've had to-day, and I was up at four without any breakfast to come to; and a pipe's a wonderful satisfier: and I'd cough whether I smoaked or not." "But why don't you go in?" I repeated. "Sure, let 'em go. They've most of 'em got work to do, and want to be off, whilst I've got none, nor can't get anny." "How do I live?" he continued, in answer to a further question. "Well, I picks up a few pence sometimes, and my wife goes out charing. She's luckier than I am, and has two or three days a week reg'lar." So communicative was he that I had little compunction in asking him how he managed to scrape together his threepence a week. "Divil a threepence can I pay," he rejoined, "and when I told 'em how it was at home they gave me a for-r-m, and somebody come to our place and looked about, and said it was all right. And so it was when I came next time." His Mark-Tapley-ian good-humour under most depressing circumstances was interesting to watch. He told me how hard he had tried to get work, and of his continual non-success. He couldn't or wouldn't see the reason, though it was plain to me. He was far from strong, his conversation was frequently interrupted by a deep cough, and it did not need an M.D. to discern that before very long consumption would show its awful marks upon his visage. He himself was a fatalist, however. "The real reason of my not getting work—between you and me, sir—is this. The year began on a Friday, and Friday is unlucky, as everybody knows, and it's always been *my* unlucky day. I says to my old woman last winter—at the beginning of the year—says I, 'you'll see I shall have bad luck, and little work all this year.' 'Rubbish,' says she, 'don't talk to me'; but you see I was right. But there's one comfort," he concluded, "the blessed year's nearly out, and they don't *all* start on a Friday." And, knocking the ashes out of his second pipe—a clay about an inch long on the stem—he bade me "good afternoon," and turned, coughing with some violence, into the hospital gates.

